# Reconstruction of Exposure to *m*-Xylene from Human Biomonitoring Data Using PBPK Modelling, Bayesian Inference, and Markov Chain Monte Carlo Simulation

**DOI:** 10.1155/2012/760281

**Published:** 2012-04-08

**Authors:** Kevin McNally, Richard Cotton, John Cocker, Kate Jones, Mike Bartels, David Rick, Paul Price, George Loizou

**Affiliations:** ^1^Health and Safety Laboratory, Harpur Hill, Buxton, Derbyshire SK17 9JN, UK; ^2^Toxicology & Environmental Research and Consulting, The Dow Chemical Company, Midland, MI 48674, USA; ^3^Mathematical Sciences Unit, Health and Safety Laboratory, Harpur Hill, Buxton, Derbyshire SK17 9JN, UK

## Abstract

There are numerous biomonitoring programs, both recent and ongoing, to evaluate environmental exposure of humans to chemicals. Due to the lack of exposure and kinetic data, the correlation of biomarker levels with exposure concentrations leads to difficulty in utilizing biomonitoring data for biological guidance values. Exposure reconstruction or reverse dosimetry is the retrospective interpretation of external exposure consistent with biomonitoring data. We investigated the integration of physiologically based pharmacokinetic modelling, global sensitivity analysis, Bayesian inference, and Markov chain Monte Carlo simulation to obtain a population estimate of inhalation exposure to *m*-xylene. We used exhaled breath and venous blood *m*-xylene and urinary 3-methylhippuric acid measurements from a controlled human volunteer study in order to evaluate the ability of our computational framework to predict known inhalation exposures. We also investigated the importance of model structure and dimensionality with respect to its ability to reconstruct exposure.

## 1. Introduction

There are numerous programs, recent and ongoing, to evaluate environmental exposure of humans to chemicals, for example, EU ESBIO, COPHES, US CDC NHANES, Canadian Health Measures Survey [1–4]. Exposure assessment is relatively simple for occupational situations but more complex for the general public where exposure occurs via poorly defined exposure scenarios and multiple pathways. Under such circumstances human biological monitoring (BM or biomonitoring) can be the most reliable exposure assessment methodology as it provides an estimate of internal or absorbed dose of chemical by integrating exposure from all routes [5]. BM is the repeated controlled measurement of a chemical, its metabolites, or biochemical markers in accessible samples such as bodily fluids (e.g., urine, blood, saliva), exhaled air, and hair [6]. In risk characterisation, BM is often superior to other methods of exposure assessment, such as personal air measurements or dermal deposition assessments, because actual estimated body burden or biologically effective dose is a composite measure of the differences in individual behaviour (e.g., personal hygiene), work rate (characterised by different respiration rates), physiology, metabolism, and hence susceptibility [5]. Uncertainty in external exposure assessment due to inter- and intraindividual variability can also be reduced by using BM if the measured biomarker, either parent chemical or metabolite, is proportionately related to the ultimate toxic entity [5].

It has been proposed that the effects on public health from exposure to environmental chemicals may be better understood when the relationships between key events along the exposure-health evaluation-risk assessment continuum are established [7]. BM is one such tool that can link external exposure and biologically effective dose. Unfortunately, it is more often the case that BM data are reported without the corresponding external exposure data, which then requires definition of the relationship with biologically effective dose. “Exposure reconstruction” or “reverse dosimetry” are terms used to describe procedures for determining estimates of external exposure consistent with BM data.

There have been a number of studies in which physiologically based pharmacokinetic (PBPK) modelling and statistical techniques were used to “reconstruct exposure or dose” consistent with human BM data at both the individual and population levels [8–15]. Population-based estimates of exposure that account for human interindividual variability, both in the modelling of chemical disposition in the body and in the description of plausible exposure conditions, can be achieved using the Bayesian inference [10]. Gelman et al. [16] used a Bayesian approach as a general method of parameter estimation in PBPK models. This method was originally applied to PBPK model calibration [17–20]. Lyons et al. [10] extended PBPK model calibration to include the unique exposure for each individual as another parameter to be estimated, alongside two additional “hyper-parameters”, the mean and standard deviation of exposures at the population level, to model variability in exposure. In this way the model could be applied to interpret population-based BM data.

The use of a PBPK model is significant because all the parameters represent anatomical, physiological, and biochemical characteristics which constrain variability to within biologically plausible limits. The limits on variability bestowed by biological structure suggest that the “ill-posed” problem of reverse dosimetry can be addressed to a certain extent. The ill-posed problem refers to the situation where any number of reverse dosimetry outcomes (reconstructed exposures) are possible, for example, an unstable model where a small change in the data may lead to a large change in output of the inverse function or no unique solution, and therefore a myriad of possible solutions or no solution at all ([21] cited by Lyons et al. [10]). Instead, knowledge regarding ranges, central values, and measures of dispersion are ascribed to model parameters, which are combined with specific data from separate studies to define informative prior distributions. Therefore, the linking of a PBPK model with Bayesian inference has a number of advantages with regard to exposure reconstruction. Firstly, it is an appropriate approach for systems where tissue dose is not necessarily linearly related to external exposure [10, 22, 23]. Secondly, defining informative prior distributions around parameters converts a deterministic model to a population model. Thirdly, this combination can extract population variability and multiple routes of exposure information integrated within BM data.

The use of a PBPK model to link BM to external exposures has already been described as significant. However, an aspect of exposure reconstruction that has not yet been adequately explored is whether any particular model is an adequate representation of the biological system it is built to emulate. If there are inadequacies in the PBPK model, then the exposure estimates will be wrong. By using data generated from a laboratory study where both the BM outputs and the exposure are known, exposure can be treated as an unknown variable to be estimated from the data, which allows the PBPK model to be evaluated and any inadequacies to be addressed. Whilst comparable data from laboratory-based studies are not a prerequisite for population-based modelling, indeed for chemicals with adverse health effects such data will not be available, human volunteer studies provide much richer data than will be available for population-based modelling. Within this environment it is possible to study how contextual information about individuals, in addition to samples, improves the results of dose reconstruction, the effect of interindividual variability on biomarker profiles can be studied, and the use of a PBPK model alongside new data streams, such as the exhaled-breath measurements used in this study, can be validated. The results from controlled laboratory-based studies for a variety of chemicals and exposure scenarios could inform improvements in population-based modelling.

In this study we used a PBPK model to evaluate the reconstruction of inhalation exposure to *m*-xylene from experimental BM data obtained in a controlled human volunteer study. Biomonitoring data comprised of timed measurements of venous blood and exhaled *m*-xylene and urinary 3-methylhippuric acid (MHA). In addition, we investigated the reconstruction of inhalation exposure when using the individual volunteer anthropometric measurements of body mass, body fat mass, resting alveolar ventilation rate, urine flow, urine creatinine concentration, and blood:air partition coefficient in addition to BM data. We also investigated the use of global sensitivity analysis [24] to reduce the computational cost of reverse dosimetry which, depending on the selected model output, is achieved by setting unimportant parameters of the PBPK model to central estimates.

## 2. Materials and Methods

### 2.1. Volunteers

The UK Health and Safety Executive Research Ethics Committee approved the study. Volunteers, who all were Health and Safety Laboratory staff, provided written informed consent before participating. Eight volunteers, 7 male and 1 female (aged 29 to 54) (Table 1), took part, were in good health at the time of the study, did not suffer from respiratory disease, and were not on any medications. Medical assessments were made immediately before the start and at the end of each experiment, to ensure that each volunteer was fit to participate and then to be discharged, respectively. The medical supervisor was present throughout the exposure period. All volunteers were asked to refrain from alcohol consumption for at least 72 h before entering the study. Body mass, height, body mass index (BMI), mass of body fat, resting minute volume, mean urine flow, urinary creatinine concentration, and *m*-xylene blood : air partition coefficient for each volunteer were measured (Table 1). Body fat was estimated using a bio-electrical impedance analyser (Bodystat 1500 Ltd., Isle of Man, UK) and by skinfold thickness measurements (Holtain callipers, Holtain Ltd, Crymych, UK). The value for mass of body fat used was the mean of the two techniques. Resting minute volume was measured using a Morgan Medical Pulmolab TF 501 apparatus at the Respiratory Function Unit of the Royal Hallamshire Hospital, Sheffield. Alveolar ventilation rate was assumed to be 70% of minute volume.

### 2.2. Chemicals

*m*-xylene (99%) and 3-methylhippuric acid (MHA) (98%) were obtained from Aldrich Chemical Co. (Dorset, UK). All other chemicals used were reagent grade or higher.

### 2.3. Exposure Protocol

Exposures were performed in the Health and Safety Laboratory Controlled Atmosphere Facility (CAF), a purpose built room 8 m^3^ in volume. Purging *m*-xylene-filled bubblers with compressed air into the CAF generated atmospheres of *m*-xylene vapour. The atmospheric concentration within the CAF was monitored continuously with a Miran infrared spectrophotometer (calibrated by an internal, closed-loop system) and by gas chromatography (Varian 6000, with a 0.05 m %0.5 mm i.d., with 5% OV10, 100–120 mesh Chrom CHP packing; injector temperature 120°C, N_2_ carrier gas flow rate 40 mL min^−1^, oven temperature 60°C) with flame ionisation detection (detector temperature 200°C, H_2_ flow rate 25 mL min^−1^, air flow rate 300 mL min^−1^, calibration using a gas sampling valve against a standard atmosphere). The CAF temperature was maintained at 25°C and 30% humidity for all experiments. 

Groups of 4 volunteers were exposed for 4 h on two separate occasions to a target concentration of 40 ppm *m*-xylene vapour. The actual exposure concentrations for the duration of the experiments were measured at 39.0 ± 3 and 37.0 ± 2 ppm, respectively.

### 2.4. Biomonitoring Data (BM)

Blood (CV) and exhaled alveolar (CXPPM) *m*-xylene and urinary MHA (C_urine_) concentrations were measured. Volunteers provided blood samples from the antecubital vein via an indwelling soft cannula. Blood samples were stored (48 hours maximum) at 4°C as whole blood until analysed. Blood samples were assayed in duplicate as follows. A 250 *μ*L sample was added to 750 *μ*L of H_2_O in a 10 mL headspace vial, which was capped with a PTFE-lined rubber septum. The sample was then incubated and continually stirred at 65°C for 10 minutes. A one mL headspace aliquot was taken using a warmed (75°C) gas-tight syringe (Fisons HS800 headspace sampler) and analysed by gas chromatography (Carlo Erba GC8000; column BP-5, 25 m % 0.32 mm i.d., 5 *μ*m film) and mass spectrometry (Fisons MD800 MS) operating in selected ion monitoring mode using positive electron ionisation (*m/z* [M^+^] 106). The limit of detection of the assay was 0.1 *μ*mol/l with intra- and interassay coefficients of variation of 5 and 10%, respectively.

End tidal breath samples (alveolar air) were taken and analysed according to the method of Dyne et al. [25] described previously [26]. 

Urine volume was recorded and samples were stored at −20°C until analysed. The major metabolite, MHA, was measured in the urine to assess the rate of biotransformation and elimination of *m*-xylene. A 0.5 mL sample of urine was mixed with 0.5 mL methanol and analysed by HPLC (Hewlett Packard 1050 Series, with autosampler, pump, degasser; column 3 *μ*m ODS, 100 % 4.6 mm) with a mobile phase of 0.1% acetic acid:methanol (85 : 15 with gradient elution), using diode array detection at a detection wavelength of 230 nm. The limit of detection of the assay was 40 *μ*mol l^−1^ with intra- and interassay coefficients of variation of 2 and 5%, respectively. Creatinine concentration was measured by using a Cobas Mira (ABX France) and an automated alkaline picrate method [27]. The coefficient of variation for intraday analysis was 1.5% and for interday analysis was 3% at 6 mM. 

**Figure 1:**
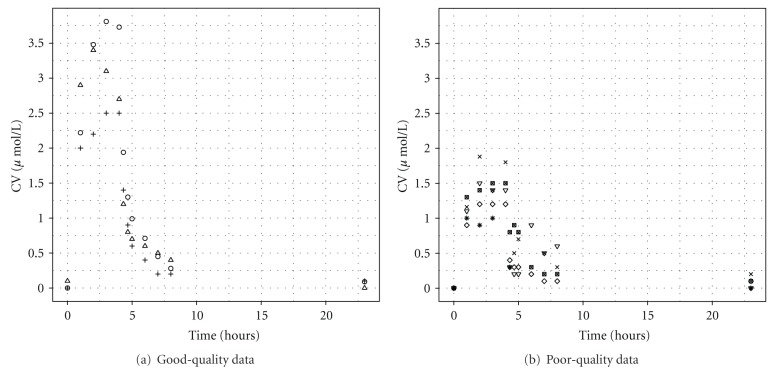
(a) Venous blood concentrations of *m*-xylene. Data from three volunteers were prepared and measured on a different day than other four. This set of data has the expected appearance and was considered acceptable for use in reverse dosimetry. (b) Venous blood concentrations of *m*-xylene. Data from four volunteers were prepared and measured on a different day than other three. This set of data does not have the expected appearance and was considered unacceptable for use in reverse dosimetry.

Venous blood samples were taken at 0, 1, 2, 3, 4, 4.33, 4.67, 5, 6, 7, 8, and 23 hours. The blood data were separated into two sets corresponding to measurements made on different occasions to investigate the importance of data quality in reverse dosimetry (Figures 1(a) and 1(b)). The analysts explain that blood data deemed unreliable may be due to imperfect sealing of sample vials leading to losses. This explanation is plausible when compared to the appearance of the exhaled breath and urine data for all volunteers, which are qualitatively similar. Also, the peak CV concentrations of the reliable data are quantitatively and qualitatively comparable with data from similar human volunteer studies [28, 29]. 

**Figure 2:**
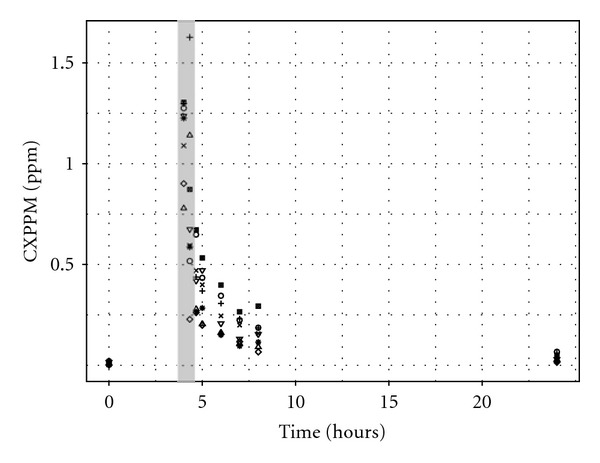
Exhaled *m*-xylene. Data from eight volunteers used in reverse dosimetry. The data points enclosed within the grey bar were excluded from the final exposure reconstruction simulations.

**Figure 3:**
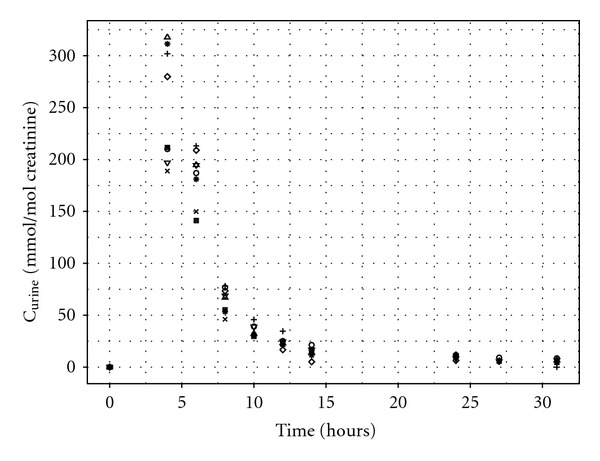
Urinary 3-methylhippuric acid (MHA). Urinary excretion of MHA expressed against creatinine for eight volunteers used in reverse dosimetry.

Exhaled air samples were taken at 0, 4.017, 4.33, 4.67, 5, 6, 7, 8, and 24 hours (Figure 2). Urine samples were taken at 0, 4, 6, 8, 10, 12, 14, 24, 27, and 31 hours (Figure 3). 

Anthropometric measurements for each volunteer are listed in Table 1.

### 2.5. The PBPK Model

**Figure 4:**
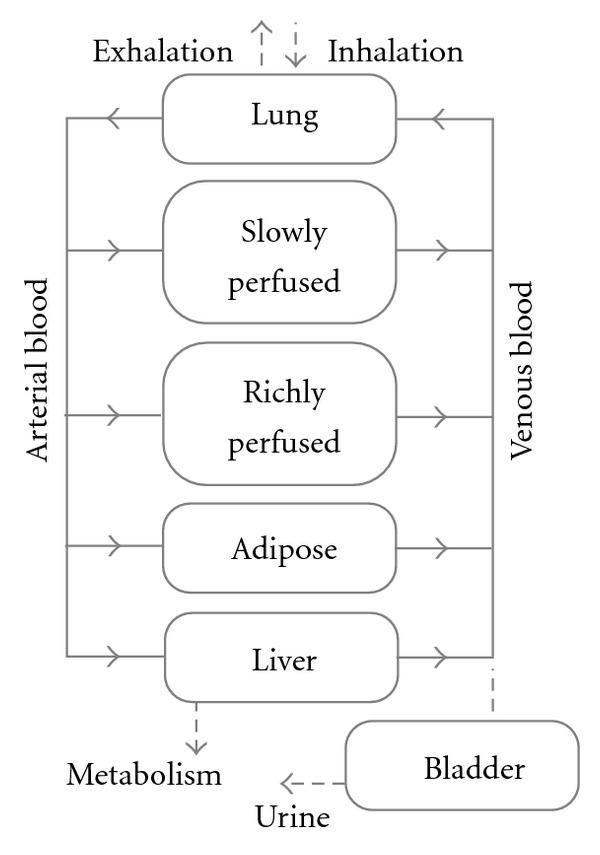
Schematic of the PBPK model for *m*-xylene with a bladder compartment, to simulate fluctuations in the concentration of the main metabolite, methylhippuric acid.

A human PBPK model that includes a bladder compartment to simulate fluctuations in metabolite concentration in urine caused by micturition [30] was adapted to study the inhalation pharmacokinetics of *m*-xylene. Liver, adipose, richly and slowly perfused tissues, and the bladder represent the body (Figure 4). The model parameter abbreviations and point values, which are similar to previous models, are listed in Table 2 along with the distributions used in the sensitivity analysis (SA) [26, 31]. Exhalation, metabolism, and renal excretion were the routes of elimination. The concentration of unbound MHA, the main metabolite of *m*-xylene in the blood, assumed to be equivalent to the concentration of compound flowing through the kidney was described by the following equations:

(1) $$\begin{array}{c} \frac{d\left({\text{MHA}}_{B}\right)}{dt}=\left(\text{MRL}i\times \frac{\text{M}{\text{W}}_{\text{MHA}}}{\text{M}{\text{W}}_{xyl}}\right)-\left({\text{MHA}}_{B}\times K_{1}\right),\\ \begin{array}{c} \frac{d\left({\text{MHA}}_{U}\right)}{dt}={\text{MHA}}_{B}\times K_{1},\\ \text{MRL}i=\frac{V_{\max}\times \text{CVL}i}{K_{M}+\text{CVL}i},\\ {\text{C}}_{\text{urine}}=\frac{{\text{MHA}}_{U}}{\text{Vo}{\text{l}}_{U}\times \text{CRE}}, \end{array} \end{array}$$

where MRL*i* is the rate of metabolism of *m*-xylene to MHA in the liver, MW_MHA_ and MW*_xyl_* are the molecular weights of MHA and *m*-xylene, respectively, MHA*_B_* is the amount of MHA in the blood, *K*_1_ is a first-order elimination rate constant describing removal of MHA*_B_* from the blood to the urine, *V*_max⁡_ is the limiting rate and *K*_*M*_ is the Michaelis-Menten constant for hepatic metabolism of *m*-xylene, CVL*i* is the hepatic venous effluent concentration of *m*-xylene, MHA*_U_* is the amount of MHA in the urine, Vol*_U_* is the volume of urine in the bladder, and CRE is the concentration of creatinine. The concentration of MHA in the urine was expressed in mmol/mol creatinine. To imitate micturition, the bladder is assumed to fill with urine at a constant (but adjustable) rate and empty at discrete time intervals (when the volume of urine reduces to zero). This enables comparison to be made between model predictions and experimental observations with timed sampling in human volunteer studies [30]. 

The Michaelis-Menten constant *K*_*M*_ and the *in vitro V*_max⁡_ for hepatic metabolism of *m*-xylene were obtained from the literature [32]. *In vitro*-*in vivo* extrapolation of *V*_max⁡_ was obtained by multiplying the *in vitro* value by a human hepatic microsomal protein yield (MPY) of 32 mg g^−1^ wet weight liver and the mass of liver (*M_Li_*) (g) [33, 34]:

(2) $$in\;vivo\;V_{\max}=in\;vitro{\;V}_{\max}\times \text{MPY}\times M_{Li}.$$

### 2.6. Parameter Distributions

Anatomical and physiological parameter distributions used for SA and MCMC simulations listed in Table 2 were obtained from the freely available web-based application PopGen, which is a virtual (healthy) human population generator (http://xnet.hsl.gov.uk/popgen/). A human population, comprising 50% male and 50% female, white Caucasians, age range 16–65, height range 140–200 cm, body mass indices 18.5–30, was generated to encompass the characteristics of the volunteers that took part in the study described below. In PopGen, organ masses and blood flows are determined for virtual individuals from both *a priori* distributions of anthropometric parameters such as body mass, height, and body mass index and measured data from existing studies. The algorithms were derived and evaluated by Willmann et al. [35]. 

No distributions were available for the partition coefficients (PCs) *Prpda*, *Pspda*, *Pfaa*, and *Plia*. Therefore, uniform distributions were assigned and the ranges set were considered reasonable assumptions. *VspdC* and *VrpdC*, the masses of the slowly and rapidly perfused tissues respectively, were not included in the SA because they are aggregated compartments from which organs and tissues are subtracted when discretely defined during model building. The model was re-parameterised as proposed by Gelman et al. [16] to ensure that mass balance and blood blow constraints were not violated. 

The mean value for *K*_1_, the first-order elimination rate constant describing removal of MHA*_B_* from the blood to the urine, was estimated by simulating the postexposure urinary excretion of methylhippuric acid following exposures at 1–10, 11–20, 21–30, and 31–40 ppm [36]. The four datasets were digitised and *K*_1_ estimated using the quasi-Newton algorithm within acslX Libero. The mean value for *K*_1_ was used in all simulations as sensitivity analysis demonstrated the model output; in this case urinary excretion of methylhippuric acid was relatively insensitive to this parameter.

### 2.7. Sensitivity Analysis

The extended Fourier amplitude sensitivity test (eFAST) for the quantitative sensitivity analysis (SA) of model parameters and the presentation of SA results as Lowry plots have been described previously [24]. The sensitivities of CV and CXPPM at the 3- and 5-hour time points within the distribution and elimination phases, respectively, and at 5 and 8 hours for C_urine_ in the early and latter urinary elimination phases are reported. Sensitivity analysis results were computed on a much finer timescale. However, the two time points selected for reporting were broadly representative of the SA results: from 0 to 4 hours absorption into the body and the period after 4 hours elimination from the body for CV and CXPPM, 4 to 8 hours “rapid elimination,” and after 8 hours “return to baseline” for C_urine_.

### 2.8. Calibration

A distribution of plausible exposures was achieved through calibration of the sensitive parameters of the PBPK model using the human volunteer data, a process referred to as reverse dosimetry. Specifically, this required a comparison of the time-varying model predictions of concentrations of substance in blood (CV), breath (CXPPM), and urine (C_urine_) with measurements in these media. 

The output from the PBPK model was represented by

(3) $$\mu_{ij}=\eta \left(x_{j},\theta_{j},\lambda ,t_{i}\right),$$

where *μ*_*ij*_ is the corresponding model prediction for subject *j* at time *t*_*i*_, **x**_*j*_ represents the vector of anthropometric measurements, and *θ*_*j*_ represents the vector of unknown variables for subject *j*. Vectors **x** and *θ* represent the vectors of anthropometric measurements and unknown parameters for all subjects, respectively. Parameter *λ* represents the (presumed) unknown exposure concentration and *t*_*i*_ indexes a specific time. SA was used to simplify the PBPK model prior to attempting calibration. Individualised parameters were only ascribed to the most important (sensitive) parameters in the model, whereas parameters that the model was relatively insensitive to were assumed to be common to all subjects and fixed at central values within their plausible ranges (Table 2). As the data were obtained in a laboratory-based study, a common exposure to all participants was assumed. More intricate hierarchical models of exposure are required to account for the heterogeneity of exposure for population studies [10]. 

**Figure 5:**
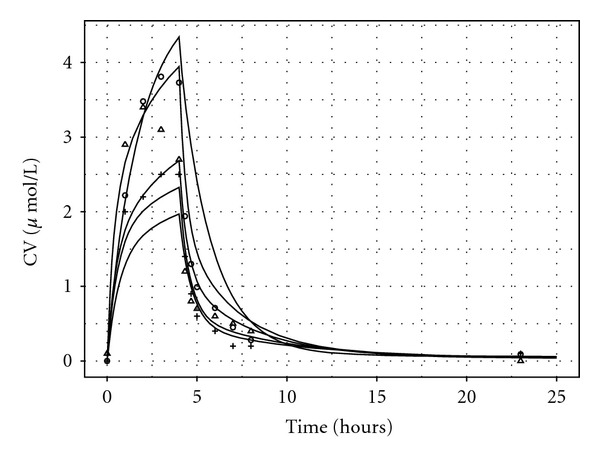
A comparison of 5 CV biomarker profiles corresponding to parameter sets sampled from the priors and reliable CV measurements.

An infinite family of parameter sets, which are inputs to the PBPK model, are defined by the (prior) probability distributions on model parameters. Each parameter set is used to overwrite the initial (default) parameters, thereby constituting a different PBPK model. In Figure 5 the reliable CV measurements and the biomarker profiles corresponding to 5 parameter sets drawn from the prior distributions of the model parameters are shown. Each PBPK model specified by a particular parameter set is unique; however, very different sets of parameters may define similar PBPK models (with respect to specific model outputs). The objective of the reverse dosimetry is to calibrate or tune the unknown parameters of the model ***θ*** and the exposure concentration *λ* such that the observational data and model predictions are in close agreement. Convergence to a unique solution was not possible (due to both measurement error and model inadequacy); however, calibration should result in a substantial reduction in the domain of the family of models that are consistent with measurements. Formally, inference was achieved through application of the *Bayes theorem.* The posterior distribution results in a narrower range of biomarker profiles than that depicted in Figure 5. 

A model that linked model predictions to observations was required. The considerations in choosing an appropriate form were that the model predictions and observations in subjects are strictly nonnegative; the magnitude of prediction errors was proportional to the magnitude of the substance; prediction errors were asymmetric, with observations much greater than predicted by the model more likely than observations far below the model predictions. These features all suggest that a model on the log scale was appropriate. Here, as in other works [10, 15, 16] a lognormal distribution was assumed for modelling the probabilistic relationship between model predictions and observations. 

Letting *y*_*ij*_ denote a measurement made on the *j*th subject at time *i*, where *μ*_*ij*_ is the corresponding model prediction, the* likelihood* based on all individuals at all measurement times is written as

(4) $$\begin{array}{cc} & \begin{array}{c} f\left(y\;|\;\theta ,\;\lambda ,\;\sigma \right) \end{array}\\ & \;=\overset{J}{\underset{j=1}{\prod }}\overset{T}{\underset{i=1}{\prod }}\frac{1}{\sqrt{2\pi \sigma^{2}}}\;\exp\left[-0.5{\left(\frac{\ln\left(y_{ij}\right)-\ln\left(\mu_{ij}\right)}{\sigma }\right)}^{2}\right] \end{array}.$$

The likelihood is a function of both parameters and data. This introduces a final unknown parameter *σ*, which is a statistical measure of the goodness of fit. 

The Bayes theorem was applied. This states that the *posterior distribution* of the model parameters (*θ*, *λ*, and *σ*) is proportional to the product of the likelihood and the prior (which is itself the product of the priors for *θ*, *λ*, and *σ*). The posterior distribution is a representation of prior beliefs (and constraints on parameters) updated by data:

(5) f(θ,  λ,  σ ∣ y)∝  f(y ∣ θ,  λ,  σ)×  f(θ)×  f(λ)×  f(σ).

Prior distributions for the *θ*_*j*_ are given in Table 2. Whilst each individual has a unique physiology, the same set of prior distributions modelled the uncertainty in each individual physiological parameters. However, whilst common prior distributions were assumed, the subjects had unique posterior distributions (informed by their personal BM data). A uniform prior on the (presumed unknown) exposure concentration between 0 and 200 ppm was assumed; the upper limit of this distribution was chosen such that the prior distribution should have no influence on the posterior distribution of *λ*. A noninformative prior (a uniform prior on the positive real line for ln *σ*) was assumed for *σ*.

### 2.9. Inference

The form of the posterior distribution (5) is complex because the PBPK model requires a numerical solution, and this could not be obtained in closed form. Inference about the parameters of the model was made using a Markov chain Monte Carlo (MCMC) sampling algorithm [37]. A single-component Metropolis-Hastings algorithm was used to draw samples. 

The samples from (5) index an updated family of PBPK models that are consistent with prior beliefs and laboratory-based observations on subjects.

### 2.10. The Simulations

The simulations were designed to investigate the precision of reconstructed exposures whilst minimising computational cost and reducing the number of influential unknown model parameters. A reduction in computational burden was achieved primarily through SA, with distributions ascribed only to those parameters with a significant contribution to model output variance. The use of the anthropometric measurements (**x**) listed in Table 1 allowed a reduction in the number of unknown and important parameters. The simulations analysed were therefore as follows.

1. Computational cost and the reliability of SA analysis were investigated by comparing the precision of two simulations where exposure was reconstructed from C_urine_. The first updated 17 parameters and the second updated the 11 most important parameters.
2. Exposure reconstruction from CV by updating the 11 most important parameters.
3. Exposure reconstruction from CV using the 11 most important parameters where eight were updated and three were individual anthropometric measurements.
4. Exposure reconstruction from suspect CV data using the 11 most important parameters where eight were updated and three were individual anthropometric measurements.
5. Exposure reconstruction from CXPPM using the 11 most important parameters.
6. Exposure reconstruction from CXPPM using the 11 most important parameters where seven were updated and four were individual anthropometric measurements.
7. Exposure reconstruction from C_urine_ by using the 11 most important parameters where six were updated and five were individual anthropometric measurements (including individual R_urine_ and CRE measured at each sampling time).

The simulations and specific anthropometric measurements used are listed in Table 3. 

### 2.11. Software

The numerical solutions to the model equations were obtained using acslX Libero version 3.0.1.6 (AEgis Technologies; http://www.acslx.com/). The M functions for eFAST and MCMC modelling included with the acslX optimum suite of tools were adapted for use in this study. Lowry plots and histograms were created using R and ggplot2 [38, 39] with additional code by Takahashi [40]. Data were digitised using Grab It! Graph Digitizer (DataTrend Software, Inc.; http://www.datatrendsoftware.com/). The computer used in this study was a Dell Optiplex 755 Intel Core 2 Duo CPU 3.00 GHz 2.00 GB RAM.

## 3. Results

### 3.1. Sensitivity Analysis

**Figure 6:**
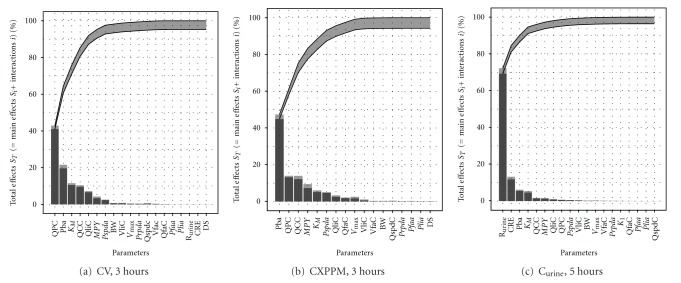
Lowry plot of the eFAST quantitative measure. The total effect of a parameter *S*_*T*_*i*__ comprised the main effect *S*_*i*_ (black bar) and any interactions with other parameters (grey bar) given as a proportion of variance. The ribbon, representing variance due to parameter interactions, is bounded by the cumulative sum of main effects (lower bold line) and the minimum of the cumulative sum of the total effects (upper bold line), (a) CV at 3 hours, (b) CXPPM at 3 hours, (c) C_urine_ at 5 hours.

Figures 6(a), 6(b), and 6(c) are typical Lowry plots for CV and CXPPM at 3 hours and C_urine_ at 5 hours used to select the most influential parameters. The total effect of a parameter *S*_*T*_*i*__ comprised the main effect *S*_*i*_ (black bar) and any interactions with other parameters (grey bar) given as a proportion of variance. The ribbon, representing variance due to parameter interactions, is bounded below by the cumulative sum of main effects (lower bold line) and above by the minimum of the cumulative sum of the total effects and one minus the sum of the main effects that were not included (upper bold line). The most influential parameters were selected by reading across from the 95% variance point on the *y*-axis to the upper bold line and then down to the *x*-axis. All parameters to the left of this point were selected and used in the dose reconstruction. The most influential parameters at the latter time points of five hours for CV and CXPPM and eight hours C_urine_ were selected in the same way (Lowry plots not shown). In Table 4 the most influential parameters at the latter time points are listed alongside those from the earlier time points. The parameters in bold are those that only become influential at the latter time points. These were added to the parameters that were influential at the earlier time points in order to ensure that prior distributions were assigned to all influential parameters across the entire time period of interest. The measured parameters listed in Table 1 are also listed in Table 3 to indicate when they were used in each simulation and are italicised in Table 4 to indicate when they contributed to variance of dose metric.

### 3.2. Computational Cost and Reliability of SA (Simulation 1)

**Figure 7:**
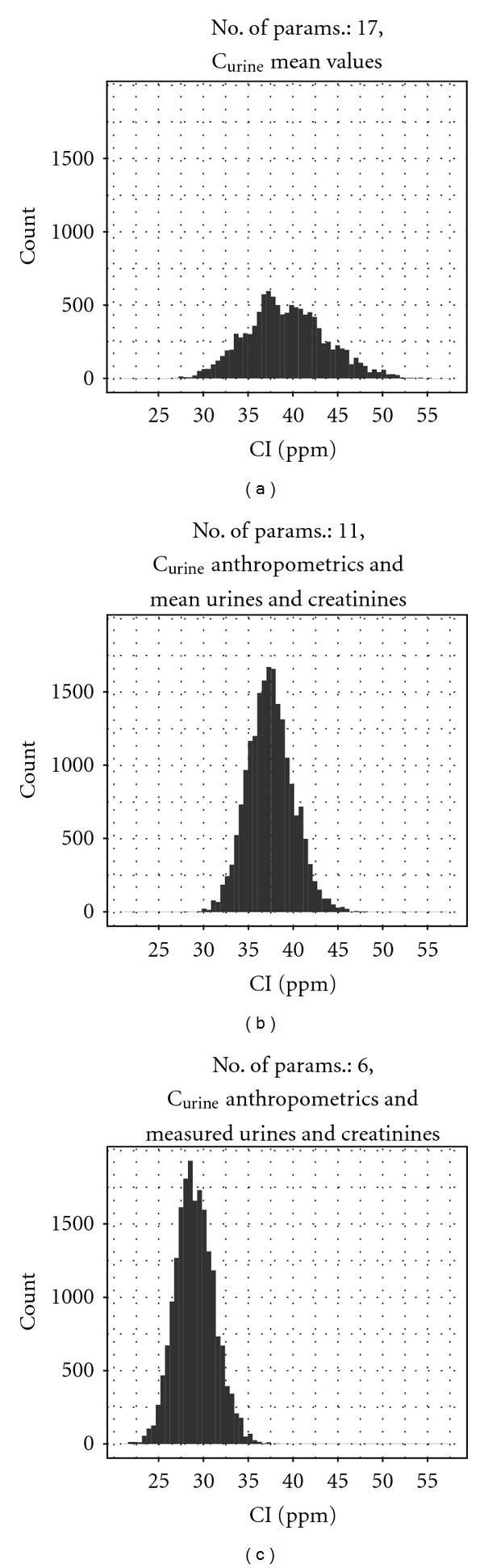
Comparison of estimated posterior distributions for 4-hour inhalation exposure to *m*-xylene. Posterior distributions were estimated by updating the entire set of parameters, most influential parameters, or by fixing the measured parameters and updating the remaining most influential: (a) C_urine_, full parameter set, (b) C_urine_, most influential, (c) C_urine_, most influential and measured spot urine production rates and creatinine concentrations.

Initially, two simulations calibrating to the observed urine measurements (C_urine_) from the volunteers were run. Both simulations employed the distributions listed in Table 2. The subset of anthropometric parameters listed in Table 1, where measurements were available for each individual, was not used in this first stage; that is, **x**_j_ was an empty set. The two initial simulations differed only in terms of the numbers of updated parameters for each individual; in the first case ***θ***_*j*_ contained 17 unique parameters for each individual; in the latter case ***θ***_*j*_ contained the 11 most sensitive parameters for each individual with the remaining parameters fixed at central values and common to all individuals (Table 3). In addition, both models also contained the unknown exposure, *λ*, and the statistical measure of fit, *σ*. In total, the models contained 138 ((17 ∗ 8) + *λ* + *σ*) and ((11 ∗ 8) + *λ* + *σ*) parameters, respectively. The objective of the comparison was to demonstrate that SA techniques could be utilized before attempting a calibration in order to reduce the dimensionality of the calculation, with only a small loss of precision. The prior distributions for each of the model parameters were common to all individuals in both simulations. Prior distributions are given in Table 2 for physiological parameters. Prior distributions for the rate of urine production and creatinine concentrations were estimated from experimental data generated in the human volunteer study (Table 1). The urine samples from the individuals allowed their unique physiological parameters to be updated, and posterior distributions were obtained for all varying parameters for each individual. Typical posterior distributions for *λ* estimated from C_urine_ are shown in Figures 7(a), 7(b), and 7(c). The mean, median, and a 95% interval for *λ* and the posterior median for *σ* are listed in Table 5. The posterior distributions for *λ* and *σ* were similar for the models that contained 138 (Figure 7(a)) and 90 (Figure 7(b)) unknown parameters respectively; however, there was a small difference in the central estimates for *λ*. This is entirely consistent with the results of the SA and demonstrates the value of appropriate SA techniques to the modeling process. Therefore, further simulations were conducted using models with prior distributions on only the most sensitive parameters. 

Due to the relatively small number of volunteers in the study, it was computationally feasible to calibrate to large numbers of individualized parameters for each subject. In a population-based study, it is likely that a balance between precision (number of individualized parameters) and computational cost would have to be achieved. For a calibration using the urine data, Figure 6(c) indicates that a calibration model using individualized parameters for the six most sensitive parameters would be adequate for capturing the majority of variance during the rapid elimination phase; however, additional parameters would be required in order to capture a large proportion of the variance in the return to baseline (slower elimination phase) period (Table 4). It is important that contributions to the variance over the full range of the measurements are considered.

### 3.3. Exposure Reconstruction from Venous Blood Biomarker (Simulations 2–4)

Two calibration models were fitted to each of the reliable CV, CXPPM, and C_urine_ datasets. The initial model did not include the measurements of the anthropometric parameters listed in Table 1 (**x**_*j*_ was an empty set), whilst the second calibration model made use of these data; therefore, there was a reduction in the vector of uncertain parameters ***θ***_*j*_ for each individual (Table 3). Calibration models making use of the unreliable CV data were also run to allow a comparison with the reliable CV data. 

**Figure 8:**
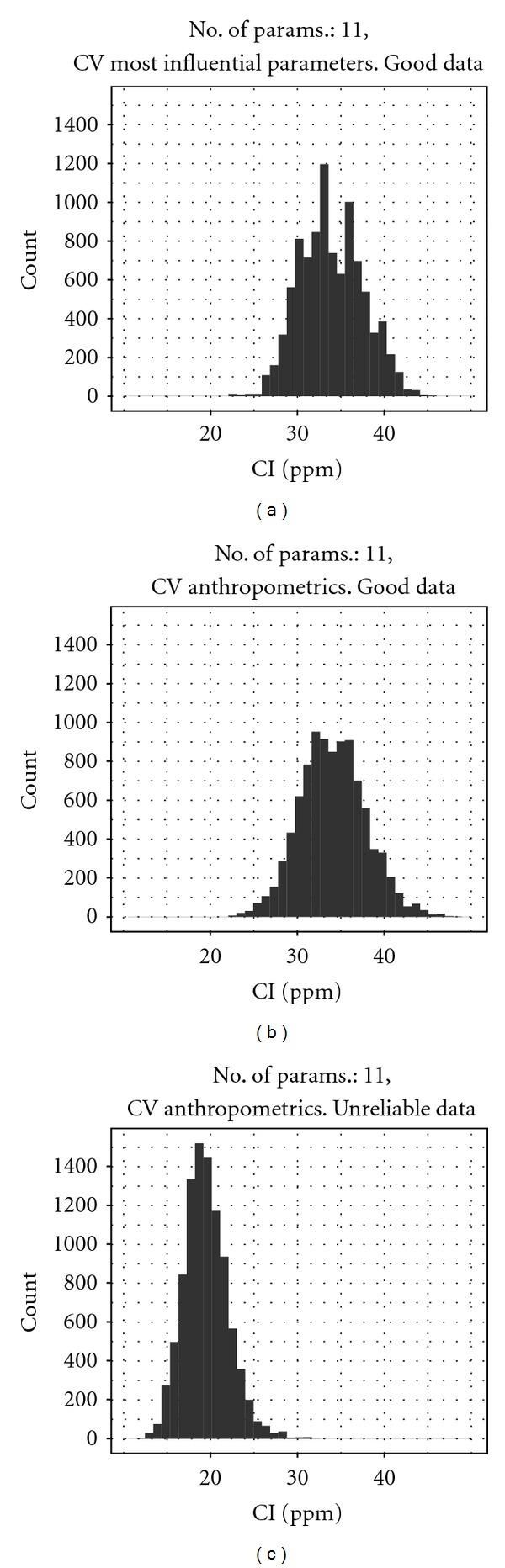
Comparison of estimated posterior distributions for 4-hour inhalation exposure to *m*-xylene. Posterior distributions were estimated by updating the most influential parameters or by fixing the measured parameters and updating the remaining most influential: (a) CV, most influential, (b) CV, fixed, measured, and remaining most influential, (c) CV, most influential, using unreliable data.

The best estimates of exposure *λ* were obtained using the reliable CV data from three volunteers (Data from the fourth volunteer were not used due to problems with taking blood samples.) although the 95% interval for *λ* was widest using the reliable CV data, which can be explained by the smaller number of volunteers and measurements (compared with data from CXPPM and C_urine_). The central estimate of *λ* was close to the target exposure of 37–39 ppm (Figure 8(a) and Table 5, reliable data, most influential), and model predictions were consistent with the observed biomarker profile (discussed later on in results). There was a small improvement in the central estimate of *λ* after making use of measured parameters **x** (comprising of QPC, Pba, and body weight) for each subject; the 95% interval for *λ* (Figure 8(b) and Table 5, reliable data, most influential including measured parameters) was shifted upward although the width of the interval was unchanged. 

The change in the posterior distribution of *λ* that resulted from using anthropometric measurements can be attributed to the correlations between *λ* and the measured parameters **x**. Whilst the exposure concentration is *a priori* independent of physical parameters, it is correlated with these parameters through the statistical model. In particular both QPC and Pba were negatively correlated (correlations between both QPC and Pba and *λ* were approximately −0.2 for each individual) with the exposure concentration in the model that treated these measured parameters as unknown. The calibration model is ill posed and cannot distinguish between the case of a low exposure and efficient transfer to the blood (larger values of QPC and Pba), the case of a high exposure and inefficient transfer to the blood (smaller values of QPC and Pba), or the continuum that exists between these two extreme cases; all are consistent with the observed data. Including measured values and thereby reducing the number of uncertain parameters in the model reduced the domain of models that were consistent with the venous blood data. There was a small increase in *σ* when including measured parameters **x**, a result of 9 less “free” parameters to describe the measurements. 

Exposure reconstruction was also attempted using unreliable CV data from four volunteers (Figure 8(c) and Table 5, unreliable data, most influential including measured parameters). The mean and median (17.4 and 17.3 ppm) of the exposure *λ* estimated from these data were approximately half of the known exposure. The 95% interval for *λ* was also very narrow. This is not a surprising finding as the CV measurements (Figure 1(b)) were much lower and more erratic compared with the reliable data (Figure 1(a)). It is clear from visual inspection of Figures 1(a) and 1(b) that the data in Figure 1(b) reach a peak at below 2 *μ*mol/l and qualitatively have an unusual profile. As mentioned previously, the analysts explain that this may be due to imperfect sealing of sample vials leading to losses.

### 3.4. Exposure Reconstruction from Exhaled Breath Biomarker (Simulations 5-6)

**Figure 9:**
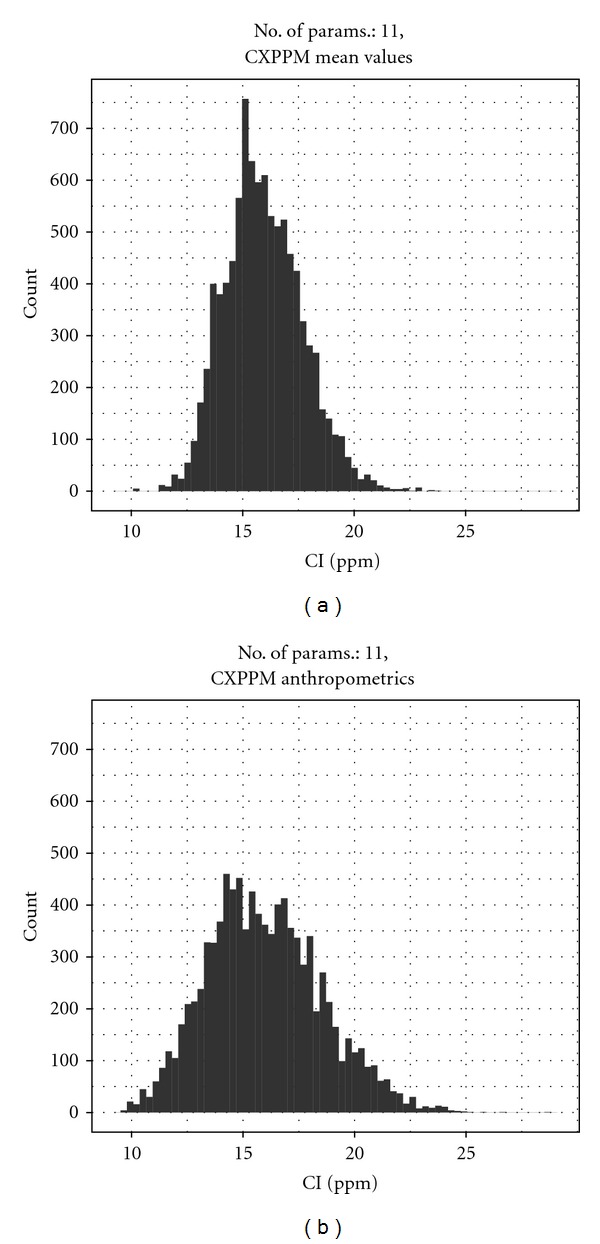
Comparison of estimated posterior distributions for 4-hour inhalation exposure to *m*-xylene. Posterior distributions were estimated by updating the most influential parameters or by fixing the measured parameters and updating the remaining most influential: (a) CXPPM, full parameter set, (b) CXPPM, most influential.

Exposure reconstruction using the entire CXPPM dataset resulted in a posterior distribution for **λ** that was concentrated on a range well below the “true” value (16.0, 15.8, 11.4, 21.4, mean and median, 2.5% and 97.5%, resp.). The simulation was repeated after excluding the measurements made at 4 hours 1 minute (i.e., one minute after exiting the exposure facility). Exclusion of these measurements is justified because, during exposure and immediately after leaving the source of exposure, the concentration of solvent in breath reflects the portion of the inhaled concentration that has not been absorbed, therefore, is not representative of circulating blood solvent levels. However, the posterior distribution for *λ* was not much improved after excluding the first measurement (Figure 9(a)) and Table 5, most influential) although still well below the target exposure. The model that included measured anthropometric parameters had an almost identical central estimate of exposure (Figure 9(b) and Table 5, most influential, including measured parameters, **x**). The error *σ* was similarly large for both of these models indicating that, even at the estimated exposures, the fit to the measurements (discussed in Section 3.6) was poor. 

### 3.5. Exposure Reconstruction from Urinary Biomarker (Simulation 7)

The first three exposure reconstructions that were attempted using the urine samples resulted in a posterior distribution for **λ** that had similar central estimates of exposure to those obtained from the reliable CV data. This is not surprising because the rate of appearance of metabolite in the urine was simulated using an empirical rate constant, *K*_1_, which in effect simply imposes a delay in the appearance of metabolite in the urine from the blood. The model including measured parameters, **x**, but with mean values for R_urine_ and CRE had a narrower confidence interval for **λ** compared with the initial models; this reduction in uncertainty can be largely attributed to R_urine_ and CRE, which sensitivity analysis showed were the most influential parameters. 

A modified PBPK model that allowed a time varying rate of urine production was fit to the data (Figure 7(c) and Table 5, most influential, including measured parameters, **x**, individual timed R_urine_, and CRE). This model had a lower central estimate for the exposure **λ** (mean and median; 29.0 and 28.9 ppm, resp.), and the narrowest 95% interval for **λ**(25.1 to 33.6). The target exposure was outside the 95% interval using the most appropriate model (that reflects real BM data, assuming that urine volumes would be recorded during sample collection). The reasons for this finding are discussed in the following section.

### 3.6. Validation and Model Criticism

A key assumption of the calibration model was of independent, normally distributed residuals (the differences between log model predictions and log (CV, CXPPM, C_urine_) measurements. Model validation was conducted to assess whether this assumption was satisfied. 

**Figure 10:**
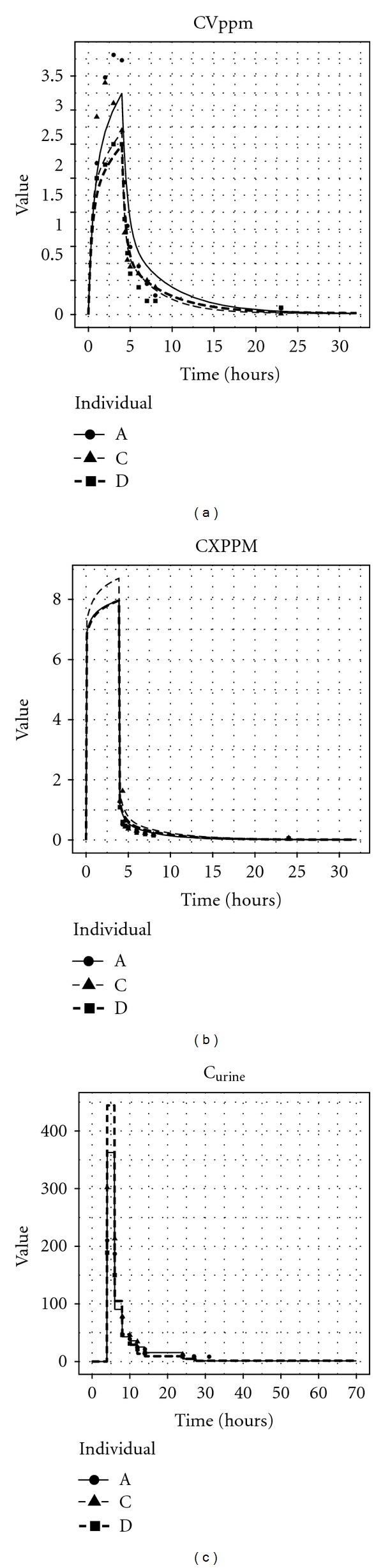
Model predictions for three volunteers from one iteration of the Markov chain and the associated measurements: (a) urine predictions and data, (b) CV predictions and data, (c) CX predictions and data.

Calibration using data from (reliable) CV measurements satisfied this assumption. Model predictions, from one iteration of parameters from the MCMC algorithm and corresponding measurements for three volunteers (A, C and D), are shown in Figure 10(a). Given that the PBPK model could predict the observed biomarker profile suggests that the model adequately describes the inhalation and subsequent excretion of *m*-xylene from the blood. 

For CXPPM the model assumption of normally distributed residuals was not satisfied. 

Predictions from the PBPK model from one iteration of parameters from the MCMC algorithm and corresponding measurements for three (representative) volunteers are shown in Figure 10(b). The figure demonstrates that the model was unable to replicate the very rapid decay of *m*-xylene in breath samples; the PBPK model proved to be inconsistent with measurements for any combination of model parameters. When using the data from CXPPM, the MCMC algorithm converged to a stationary distribution that minimized the overpredictions from the model and clearly the posterior distribution for *λ* is an unreliable estimate of exposure. The biomarker profiles from the eight volunteers were consistent and indicated that the measurements were reliable; therefore, the issue is with the PBPK model. The information from CV measurements gives some context to interpret this result as these data highlight that the model can adequately describe the uptake, and indeed the elimination of *m*-xylene, from the blood. Therefore, the PBPK model currently lacks some biological detail for describing the mechanism of exhalation of *m*-xylene. This is an area of ongoing research. 

Whilst the estimates of exposure using the C_urine_ measurements and the PBPK model with individualised urine volume and creatinine measurements were close to the target exposure, the assumptions of the calibration model were not satisfied. Model predictions, from one iteration of parameters from the MCMC algorithm and corresponding measurements for three volunteers, are shown in Figure 10(c). The model proved a reasonable fit to measurements taken between 8 and 14 hours but over-predicted the initial elimination (4 and 6 hours) and under-predicted the concentrations the following day (at 24, 27, and 30 hours). The large difference in results from the model using the individualised urine volume and creatinine measurements compared with the other PBPK models based upon the urine measurements resulted from the former model providing a better fit to the measurements at 6 and 8 hours, whereas the latter models were a better fit to measurements at 24, 27, and 30. Given that the uptake of *m*-xylene to the blood is adequately described, this suggests the model lacks biological detail in the elimination of substance in urine. 

Elimination of *m*-xylene in urine has been studied over a 60-hour period [36]. The data from this study are consistent with our own laboratory based study in showing that there is an initial decay to a nonzero value (the first 10 hours or so after exposure has ceased) and a much slower elimination of substance (taking in excess of 2 days) that follows. The model may require a “deep tissue” compartment to simulate the slower redistribution from fat and other tissue storage compartments [41].

### 3.7. Additional Results

A model using the reliable CV data with just the exposure concentration varying and all parameters fixed at central values was run for comparison (Table 5, all parameters fixed at central values). Such a model does not take human variability into account; the measurements are all, in effect, on the “mean human” with parameters set at the central values listed in Table 2. The result was a much lower central estimate of **λ** (24 and 23.8 ppm, mean and median, resp.), a narrower 95% confidence interval for the exposure (21–29 ppm), and a larger median for *ρ* (0.55). These latter two results are consistent with expectations. The lower range of exposures resulted from the 3 volunteers not being representative of the “mean human” (in particular with respect to PBA and QPC). 

This admittedly unusual result demonstrates that it is important to recognise and model all known sources of uncertainty. However, the large difference in the 95% confidence intervals between this and our fitted models does suggest that the prior distributions for the model parameters were too wide; the subjects in our study were more homogeneous than the general population. More precise results would have been obtained if prior distributions based upon the study population had been adopted. In future work a greater emphasis will be placed on understanding and properly defining the study population.

## 4. Discussion

In this paper it was demonstrated that SA techniques could be used to reduce the dimensionality of the calibration problem with a relatively small loss of precision. The use of SA in this context is consistent with good modelling practice, with SA used as an integral part of the modelling process [42–45]. SA demonstrated that exposure reconstruction using urine data requires a greater number of individualised parameters in the PBPK model than either exhaled breath or blood. This is not surprising as the PBPK model needs to describe uptake, distribution, metabolism, and excretion in order to reconstruct exposure using urine measurements, and excretion from the body (in urine) takes in excess of 30 hours. More parameters are required to describe this process, with some parameters only becoming important at later periods (Table 4). In comparison the SA results for blood and breath models were more consistent, the same parameters were important throughout the full time-scale, although the relative importance of parameters did change, parameters governing uptake dominated during exposure, and these became less important after exposure ceased. It is important that the SA of the PBPK model covers the full period where measurements (for comparison with the model) are available, so that the important parameters for model calibration using the available data are identified. 

The results from SA did inform one substantial improvement in the description of urination in the PBPK model. It was noted that the model was especially sensitive to R_urine_, the rate of urine production, and to a lesser extent CRE. The initial PBPK model represented these as constants (either unknown constants represented by probability distributions or as constants estimated from measurements, depending upon the model) for each individual. However, the experimental data revealed that there were substantial variations for each individual in both the rate of urine production and CRE over the 31-hour period of the study. The PBPK model was modified to allow time varying rates of R_urine_ and CRE. Here it can be seen that SA techniques can be used to inform model development as well as to reduce complexity. Also, the results of SA can help inform the prioritisation of resources and effort in the generation of good-quality data. 

The aim of this work was not to estimate the most precise exposure for the subjects using all available measurements; rather the aim was to reconstruct exposure using a single measurement series and account for all significant uncertainties. This work can be viewed as an intermediate step between laboratory- and population-based studies. Models have been independently fit to breath, blood, and urine measurements, as these are a closer approximation to the data from a population-based study. However, whilst the models were independently fit, a more complete picture of the inadequacies of the PBPK model is obtained by interpreting the results collectively. 

Looking into the future, the greatest challenge is to obtain estimates of a variable exposure concentration from biological monitoring data available from individual “spot samples,” typically at the end of a shift for workplace exposures or at random times for environmental exposures. The appropriate extension to the calibration model compared with that used for data from our controlled laboratory study is straightforward. Each individual will have a unique exposure; a hierarchical model of exposure (Lyons et al., [10]) can be used to obtain a central estimate and an estimate of the variability in personal exposures. A greater challenge is that in general a single sample per individual may be available, *and* the time between the completion of exposure and the production of a urine sample will vary. As this study has shown, there is a strong correlation between the length of time after exposure and the sample concentration for nonpersistent and semipersistent chemicals (Figures 1, 2, and 3). Based upon the data available from biological monitoring, it will be very difficult to isolate personal exposures, after accounting for both biological variability *and* time after exposure. An intricate calibration model will be required. This is a major challenge for future work, although empirical statistical models may prove a useful tool. One option might be a two-phased approach: in the initial phase, a nonlinear mixed-effect model could be used, with a mean corresponding to a PBPK model containing the unknown exposure concentration as the sole unknown parameter and systematic differences between humans modelled by random effects. The posterior distribution for the exposure concentration from this first phase could provide an informative prior for the exposure concentration in more detailed PBPK models. The precision of exposure reconstruction would undoubtedly be improved if some contextual information (relating to the time since the last “significant” exposure) accompanied each sample. 

Although this work has focussed on the calibration problem with respect to the exposure concentration, the data allow all the model parameters to be updated in light of the data. This includes updating the parameters for each (experimental) subject for quantities where population variability is well characterised (such as organ sizes and blood flows) and quantities about which there is considerable uncertainty in the general population. Some of the partition coefficients fell into this latter category; uniform distributions with wide support were used as the prior distributions for the partition coefficients *Pspda*, *Prpda,* and *Pfaa*. In principle the data would allow individualised partition coefficients to be estimated for each of the subjects. However, when there is uncertainty about variability in the general population, it is appealing to structure a model in order to learn about population variability; this can be achieved using a hierarchical model that incorporates a population mean and standard deviation into the calibration model. However, in this work the SA indicated that the model was insensitive to these uncertain partition coefficients, and these were therefore fixed at central values in the model. 

Initial models using CV, CXPPM, and C_urine_ data ignored the subset of anthropometric parameters (listed in Table 1) that were available for each individual, and it was possible to compare the posterior distributions for these parameters with the known values. The posterior distributions for these parameters were similar to the priors: the data from subjects did result in some small changes; however, due to the large number of uncertain parameters for each individual, coupled with an unknown exposure, the changes were modest. For body weight (BW), the fat mass (VfaC), the blood : air partition (Pba) coefficient, and alveolar ventilation (QPC), in general the posteriors moved toward the known values (compared with the priors), although there was only a modest reduction in uncertainty compared with the prior. This is consistent with expectations; as the PBPK model is ill posed, a strong convergence to the known values for these parameters could not be achieved. 

Given that the priors were similar to the posteriors, it is important to question the value of a fully Bayesian analysis using an MCMC algorithm that updates the parameters for each individual compared with a Monte-Carlo (MC) algorithm that accounts for interindividual differences by sampling from the priors. In this case a comparison of prior and posterior distributions for individual parameters is overly simplistic. A priori all parameters are independent, whereas parameters are correlated in the posterior distribution. This means that the multidimensional posterior is a very different shape to the multidimensional prior, even though the marginal distributions are similar. Specifically, a smaller domain of PBPK models is consistent with the observed data after calibration. In cases where fewer data are available for calibration, MCMC will not offer the same improvements over MC sampling. 

Throughout the paper we have used the terms *uncertainty* and *variability*, which are important and related concepts. There are numerous and wide ranging uncertainties in many modelling scenarios. O'Hagan and Oakley [46] identify the main components of uncertainty for computer models as parameter uncertainty, model inadequacy, residual variability, and code uncertainty. The various uncertainties in a model can be described as aleatory uncertainties, which arise from inherent variability or randomness in systems, and epistemic uncertainties, which arise due to imperfect knowledge. Oberkampf et al. [47] noted that a variety of terms in the literature have been used to describe these two classes of uncertainty; the terms irreducible (aleatory) and reducible (epistemic) uncertainty have perhaps the greatest clarity. Model inadequacy is a reducible source of uncertainty, residual variability may contain both reducible and irreducible uncertainties, and parameter value uncertainty is generally irreducible [46]. Probability can represent both reducible and irreducible sources of uncertainty. 

Other authors use the term variability in place of alleatory uncertainty; however, we feel that this lacks clarity [48]. Variability is certainly closely related to alleatory uncertainty and can be both a cause of *and* a result of alleatory uncertainty. This can be seen in parameter value uncertainty, where interindividual differences (variability) between humans (partition coefficients, blood flows, organ masses, etc.) are represented by probability. There is parameter value (alleatory) uncertainty in a PBPK model for any given human due to interindividual differences; the unique parameters for any individual are *unknown*; only variation across the population is known. The net result is an increased variability in the outputs of that model, which arise from the parameter value uncertainty. Epistemic uncertainty also results in increased variability in model outputs. 

In conclusion, the integration of PBPK modelling, global SA, Bayesian inference, and Markov chain Monte Carlo simulation is a powerful approach for exposure reconstruction from BM data. The use of global SA techniques could be used to reduce the dimensionality of PBPK models with a minimal loss of precision and with consequent savings in computational cost. Also, the use of SA in the model building and calibration phases is consistent with good modelling practice. However, the precision of posterior estimates of exposure is exquisitely dependent upon the ability of the PBPK model to characterise the chosen biomarker, which in turn is also exquisitely dependent upon the extent of biological detail captured in a PBPK model. Further work, on the level of detail required to satisfactorily describe renal elimination and exhalation of volatile biomarkers of exposure, is required.

## Figures and Tables

**Table 1:** Individual volunteer parameters.

Volunteer	Age	Body weight (BW) (kg)	Height (m)	BMI (kg/m^2^)	Mass of body fat (VfaC) (% (BW))	Resting alveolar ventilation rate (QPC) (l/hr)	Urine flow (R_urine_) (l/hr)	Urinary creatinine (CRE) (mmol/l)	*m*-Xylene blood : air partition coefficient (Pba)
A	54	79	1.68	28.00	0.218	383.3	0.070	14.8	15.1
B	51	61.5	1.78	19.32	0.192	409.3	0.125	7.30	16.8
C	47	89	1.91	24.40	0.169	477.5	0.090	14.9	11.4
D	48	85	1.75	27.80	0.263	362.7	0.091	13.0	18.0
E	29	76	1.85	22.20	0.130	327.2	0.088	12.5	26.5
F	25	76	1.83	22.70	0.162	352.6	0.055	14.8	20.2
G	41	75	1.70	26.00	0.179	462.2	0.076	12.8	—
H	29	68	1.70	23.50	0.299	348.6	0.074	10.2	21.6
Mean		76.2	1.78	24.24	0.202	390.4	0.083	12.5	18.5
SD		8.73	0.08	2.95	0.056	54.89	0.021	2.70	4.90
CV		0.115	0.05	0.122	0.277	0.141	0.247	0.212	0.26

**Table 2:** Anatomical, physiological, and kinetic constants and parameters used in the PBPK model.

Parameter	Abbreviation	Value	Distribution
Molecular mass *m*-xylene (g/mol)	MW_xyl_	106.17	—
Molecular mass MHA (g/mol)	MW_MHA_	193.2	—
Body mass (kg)	BW		Normal BW~N(76.2, (8.73)^2^)
Vascularised tissue (proportion of body mass)	VT	0.91	—
Cardiac output (L h^−1^ BW^−0.75^)	QCC		Normal QCC~N(13.8, (2.5)^2^)
*Metabolism (Liver) *			
*In vitro* Michaelis constant (mMol L^−1^)	*K_M_*		Normal K_M_ ~N(11.8, (1.4)^2^)
*In vitro* maximum rate of metabolism (pmol min^−1^ mg^−1^ microsomal protein)	*V*_max⁡_		Normal *V*_max⁡_ ~ N(895, (68)^2^)
Microsomal protein yield per gram wet weight liver (mg g^−1^)	MPY		Lognormal ln (MPY)~N(3.7, (2.9)^2^)
*Gas exchange *			
Respiratory rate (L h^−1^)	QPC		Normal QPC~N(390.4, (54.9)^2^)
Respiratory dead space (proportion respiratory rate)	DS	0.3	—
*Partition coefficient *			
Blood : air partition coefficient	Pba		Normal Pba~N(18.5, (4.9)^2^)
Rapidly perfused	Prpda		Uniform Prpda~U(50–150)
Slowly perfused	Pspda		Uniform Pspda~U(40–80)
Adipose	Pfaa		Uniform Pfaa~U(1400–2200)
Liver	Plia		Uniform Plia~U(150–350)
*Tissue blood flow as a fraction of cardiac output*			
Rapidly perfused	QrpdC	0.48	—
Slowly perfused	QspdC	0.22	Uniform QspdC~U(0.2–0.35)
Adipose	QfaC	0.05	Normal QfaC~N(0.053,(0.003)^2^)
Liver	QliC	0.25	Normal QliC~N(0.271,(0.01)^2^)
*Tissue mass as a fraction of body mass *			
Rapidly perfused	VrpdC	0.09	—
Slowly perfused	VspdC	0.604	—
Adipose	VfaC	0.19	Lognormal ln(VfaC)~N(−1.59,(−2.88)^2^)
Liver	VliC	0.0257	Normal VliC~N(0.036,(0.01)^2^)
*Bladder compartment*			
Rate of urine production (L h^−1^)	R_urine_	0.07	Normal R_urine_ ~ N(0.083,(0.021)^2^)
Urinary creatinine concentration (mmol L^−1^)	CRE	12.5	Normal CRE~N(12.5,(2.7)^2^)
First-order elimination rate constant (h^−1^)	*K_1_*		Uniform K_1_~U(5–20)

**Table 3:** Summary of simulations.

Simulations	Number of updated parameters		
	CV	CXPPM	C_urine_
Full set (mean values)	0	—	17
Most influential	11	11	11
Most influential-**(x)**	8	7	6

	*Parameters not updated*		

Measured parameters **(x)**	QPC	QPC	QPC
	PBA	PBA	PBA
	BW	BW	BW
		VfaC	R_urine_
			CRE

**Table 4:** Model parameters accounting for 100% variance at different time points.

CV		CXPPM		C_urine_	
3 h	5 h	3 h	5 h	5 h	8 h
*QPC* ^ a^	*QPC*	*Pba*	*Pba*	R_urine_	R_urine_
*Pba*	**QspdC**	*QPC*	*QPC*	*CRE*	*BW*
KM	KM	QCC	QspdC	*BW*	*QPC*
QCC	QCC	KM	KM	*Pba*	*CRE*
QliC	*Pba*	QliC	QCC	KM	***VfaC***
MPY	QliC	MPY	**Prpda**	QCC	**Qspdc**
Pspda	Prpda	Pspda	QliC	MPY	**Pspda**
*BW*	MPY	VliC	MPY	QliC	*Pba*
VliC	Pspda	*BW*	Pspda	*QPC*	QCC
		QspdC	VliC		**Pfaa**

^a^Italicised abbreviations correspond to parameters measured for each volunteer listed in Table 1.

**Table 5:** Posterior distributions of inhalation exposure to *m*-xylene.

Biomonitoring data	Parameters updated		*m*-xylene inhalation exposure posterior distribution				Statistical measure of fit
			Mean	Median	2.5%	97.5%	Median
CV		All parameters fixed at central values (“mean human”)	24.0	23.8	21.0	29.0	0.55
	Reliable data	Most influential (Figure 8(a))	33.8	33.5	26.6	41.5	0.35
		Most influential including measured parameters (x) (Figure 8(b))	36.2	35.9	29.5	44.5	0.38
	Unreliable data	Most influential including measured parameters (x) (Figure 8(c))	19.5	19.1	15.3	25.0	0.71

CXPPM	Most influential (Figure 9(a))		16.0	15.8	11.4	21.4	0.58
	Most influential including measured parameters (x) (Figure 9(b))		15.9	15.8	13.0	19.6	0.59

**C**_**urine**_	All parameters (mean **R**_**urine**_ and CRE) (Figure 7(a))		39.3	39.1	31.2	48.7	0.356
	Most influential (mean **R**_**urine**_ and CRE)		38.7	38.4	30.7	48.2	0.349
	Most influential including measured parameters (x) (mean **R**_**urine**_ and CRE) (Figure 7(b))		37.5	37.4	32.7	42.7	0.37
	Most influential including measured parameters (x) (individual timed **R**_**urine**_ and CRE) (Figure 7(c))		29.0	28.9	25.1	33.6	0.4
